# The Usefulness of the Pressure Algometer in the Diagnosis and Treatment of Orofacial Pain Patients: A Systematic Review

**DOI:** 10.1155/2020/5168457

**Published:** 2020-06-11

**Authors:** Agata Kamińska, Bartosz Dalewski, Ewa Sobolewska

**Affiliations:** ^1^Chair and Department of Dental Prosthetics, Specialists' Dental Clinic, Pomeranian Medical University in Szczecin, Poland; ^2^Chair and Department of Dental Prosthetics, Pomeranian Medical University in Szczecin, Poland

## Abstract

**Objectives:**

The pressure pain threshold (PPT) may be an efficient approach to screen and evaluate orofacial pain. However, the results of previous PPT studies have varied greatly. The aim of this paper was to determine whether the PPT is an efficient approach for screening and evaluating orofacial pain.

**Methods:**

The search yielded 123 articles. After removal of duplicates and screening of abstracts, 32 articles were selected for further evaluation. The Cochrane Collaboration tool for assessing the risk of bias was used for the evaluation of the studies.

**Results:**

The studies covered a total of 4403 adult patients, aged 16-62, and 30 children. The studies investigated the reliability and validity of the PPT (measured by a pressure algometer) in TMD patients. The PPT was investigated in relation to headache, menstrual cycle, oral contraception, occlusal interference, and occlusal appliances. Generally, the risk of bias was low to unclear. Some structural limitations were inherent in the studies, such as small samples and short duration of the testing involved. Also, the analyzed studies lacked consistency in study design and patient management. Pressure increase values differed from 20 kPa/s to 50 kPa/s and from 0.5 kg/cm^2^/s to 2 kg/cm^2^/s. Descriptions of the PPT examination points also varied, from very precise and repeatable to a simple listing of anatomical points. The number of measurements varied from 1 to 5 at each visit. The intervals ranged from 5 seconds to 15 minutes. However, some studies confirmed that the pressure algometer is an effective tool for determining the source of orofacial pain.

**Conclusions:**

Based on the analyzed articles, the authors argue that the PPT is not an efficient approach for screening and evaluating orofacial pain. What is more, it should not be used as the only diagnostics tool for patients with orofacial pain. Importantly, however, additional factors should be considered in the future for the evaluation of the PPT, including body symmetry and posture, hormone levels and the menstrual phase in women, and the use of medications and its influence on the PPT. Further clinical trials should also be performed on the PPT, examining head and neck pain patients, with more precise study design and larger samples.

## 1. Introduction

### 1.1. Rationale

According to the definition of pain proposed by the Subcommittee on Taxonomy of the International Association for the Study of Pain, pain is a subjective sensation which is individual and depends on numerous contributing factors. Pain in the orofacial region influences everyday life, largely by limiting the ability to chew or speak, which calls for the investigation of the problem to improve diagnosis and treatment of orofacial pain patients [1]. Myofascial TMD pain is the most common diagnosis (42%) among patients reporting to the dental office due to orofacial pain [2]. It occurs as a variety of conditions that can affect the temporomandibular joints (TMJ), face, head, and cervical joints [1]. Myofascial pain is about three times more common in women than in men and mostly reported among TMD patients (45.3%), patients suffering from TMJ disc displacement (41.1%), or patients with TMJ arthralgia (34.2%) [3]. Myofascial pain is one of the most common causes of pain. The myofascial pain syndrome (MPS) is diagnosed mainly by muscle palpation. The source of the pain in MPS is the myofascial trigger points, which are localized tender regions, easily identified by palpation [1]. Diagnosing and management of orofacial pain is a complex, multifaceted, and multidisciplinary process.

The PPT may be an easy and efficient method to screen and evaluate orofacial pain [4]. The PPT is the minimum application force which induces pain. Devices of various types and designs have been used in past diagnosis and treatment; however, their diagnostic values still remain controversial. Pressure algometers are used to measure the PPT of selected muscle and bone locations.

### 1.2. Objectives

This review assesses the efficacy and usefulness of the pressure pain algometer in patients experiencing orofacial pain, on the basis of clinical trials performed in a recent 20-year period (1997-2017). Importantly, the number of such clinical trials remains limited. By the application of the PICO method (population, comparison, and outcome), the focused question was developed, namely, whether pressure algometry is an efficient approach for screening and evaluating orofacial pain. There has been no systematic review to date of the usefulness of the pressure algometer among orofacial pain patients.

## 2. Methods

### 2.1. Protocol and Registration

The protocol for this systematic review was registered in the International Prospective Register of Systematic Reviews (PROSPERO) (*registration* number CRD42017079566).

### 2.2. Eligibility Criteria

Inclusion criteria are as follows: articles published in a recent 20-year period (1997-2017), written in English, and covering human research.

Exclusion criteria are as follows: articles published earlier than 1997, not written in English, animal studies, studies with the use of medications, and articles examining receptor interactions.

### 2.3. Information Sources

**Figure 1:**
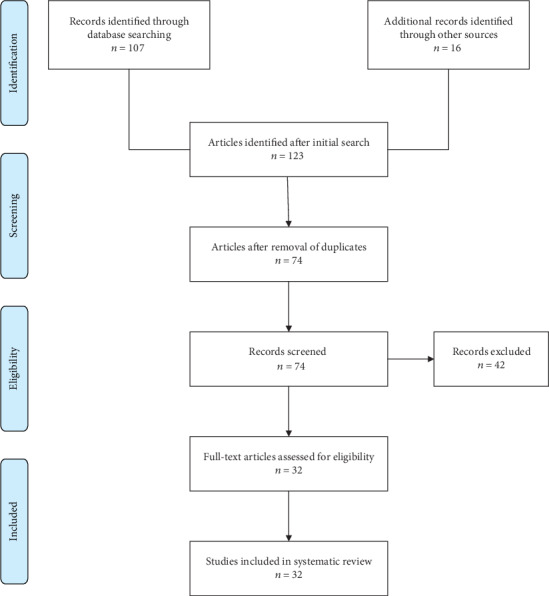
PRISMA diagram.

Literature search was performed from 1 December 2017 through 12 January 2018, covering PubMed, Dentistry and Oral Sciences Source, Web of Science, ProQuest, Scopus, Medline (EBSCO), and ScienceDirect. The selection key is presented in the PRISMA diagram (Figure 1).

### 2.4. Search Strategy

The primary search strategy involved the identification of four primary keywords and then was adapted to other databases by adding two secondary keywords. The primary keywords were pressure threshold, masseter muscle, facial pain, and tmj. The secondary keywords were temporal muscle and algometer.

*The search strategy equation for PubMed*: (“Pain Threshold”[Mesh] AND “Temporomandibular Joint”[Mesh]) AND (“Masseter Muscle”[Mesh] AND “Facial Pain”[Mesh]). Adding temporal muscle and algometer (secondary keywords) to the search phrase returned “0” articles. Thus, only primary keywords were used in this search.

*The search strategy for Scopus*: TITLE-ABS-KEY (pressure AND threshold AND facial AND pain AND tmj AND masseter AND muscle) AND (LIMIT-TO (PUBYEAR, 2014) OR LIMIT-TO (PUBYEAR, 2013) OR LIMIT-TO (PUBYEAR, 2012) OR LIMIT-TO (PUBYEAR, 2008) OR LIMIT-TO (PUBYEAR, 2004) OR LIMIT-TO (PUBYEAR, 2003) OR LIMIT-TO (PUBYEAR, 1997)). Adding temporal muscle and algometer (secondary keywords) to the search phrase returned “0” articles. Thus, only primary keywords were used in this search. Nine articles were identified with this search strategy, two of which were older than 20 years. The years specified for the search strategy are the dates of publication of the articles found. The Scopus database dictionary uses the phrase “pressure threshold” instead of “pain threshold”; thus, the keyword for the search strategy was modified.

*The search strategy for Medline*: (MH “Pain Threshold” and MH “Facial Pain” and MH “Masseter muscle” and MH “Temporomandibular Joint”). The search with the primary keywords returned 36 articles. After adding temporal muscle and algometer (secondary keywords), the search returned only one article. Thus, only primary keywords were used in this search.

*The search strategy for ProQuest*: pain threshold AND masseter muscle AND facial pain AND tmj AND temporal muscle AND algometer.

*The search strategy for Dentistry and Oral Sciences Source*: pain threshold AND masseter muscle AND facial pain AND tmj. Adding temporal muscle and algometer (secondary keywords) to the search phrase returned “0” articles. Thus, only primary keywords were used in this search.

*The search strategy for ScienceDirect*: pain threshold AND masseter muscle AND facial pain AND tmj AND temporal muscle AND algometer. The search strategy for ScienceDirect yielded 54 titles, of which 35 were articles.

### 2.5. Study Selection and Data Collection Process

In total, 74 articles were screened by two researchers (the two authors whose names appear first) according to the above criteria, excluding duplicates. When the two researchers disagreed as to the selection, the third author made the decision, whether the article should be included into the review. Ultimately, 32 studies were eligible for further review. The Cochrane Collaboration tool for assessing the risk of bias was used for clinical trial evaluation.

### 2.6. Data Items

The following keywords were used: algometer, facial pain, masseter muscle, pain threshold, temporal muscle, and tmj. The Scopus database dictionary uses the phrase “pressure threshold” instead of “pain threshold,” and the authors modified the keyword for the search strategy accordingly.

### 2.7. Risk of Bias in Individual Studies

**Figure 2:**
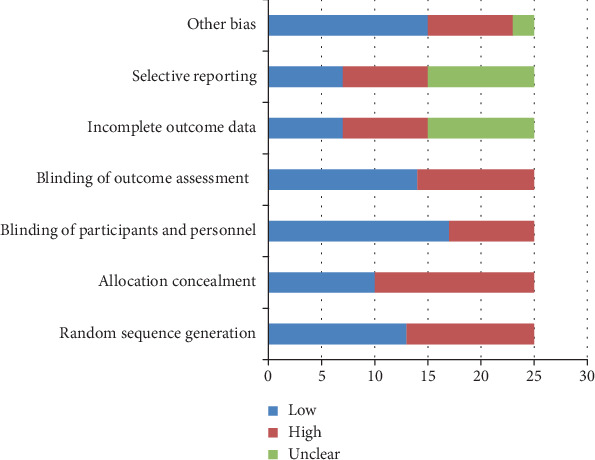
Risk of bias graph.

The Cochrane Collaboration tool for assessing the risk of bias was used for the evaluation of clinical trials (Table 1, Figure 2).

### 2.8. Synthesis of Results

A narrative synthesis of results was performed. No meta-analysis could be performed due to the heterogeneity between studies (different study designs; different algometer models; and discrepancies regarding the pressure increase rate, the number of measurements, intervals, and the presence of the control point for PPT measurement outside the facial area under examination).

### 2.9. Risk of Bias across the Studies

The GRADE approach was used for assessing the risk of bias across the studies.

## 3. Results

### 3.1. Study Selection

The initial search was performed on 10 January 2018. It identified a total of 123 articles, of which 32 full-text articles were taken for further evaluation. Of these, 25 comprised randomized controlled trials, two were OPPERA case studies, two were case-control studies, and the remaining three were research reports. The reporting of this systematic review adhered to the PRISMA Statement (Figure 1).

### 3.2. Study Characteristics

#### 3.2.1. Interventions

The studies investigated the reliability and validity of the PPT (measured by pressure algometer) in TMD patients. The PPT was investigated in relation to headache, menstrual cycle, oral contraception, occlusal interference, and occlusal appliances. Some studies compared pressure algometry and manual palpation. Others focused on factors that could change PPT values, such as physical therapy, counselling, low-level laser therapy, mechanical and electrical stimulation, orthognathic surgery, or transcutaneous electrical nerve stimulation.

#### 3.2.2. Participants

The studies included a total of 4403 adult patients, aged 16-62, and 30 children.

#### 3.2.3. Duration

The observation period varied among the studies, from 1 day to 1 year.

### 3.3. Risk of Bias within Studies

The collaboration tool for assessing the risk of bias was used for the evaluation of clinical trials (Table 1). Quality assessment of the trials demonstrated that, generally, the risk of bias was low to unclear. The low risk of bias was mostly related to group randomization, blinding of participants and personnel, blinding of outcome, and other bias. The risk of bias was mostly unclear due to incomplete outcome data and selective reporting. High risk was related to allocation concealment.

### 3.4. Results of Individual Studies

Chaves et al. observed that algometry has better intra- and interexaminer reliability than manual palpation [5]. A subsequent study by Chaves et al., comparing algometry and muscle palpation, confirmed that algometry is more effective for the examination of pain perception in widespread orofacial pain and that muscle palpation is superior for differentiating healthy controls from groups that report pain [6]. Visscher et al. revealed that the recognition of TMD pain complaints by pressure algometry was comparable to the recognition achieved by palpation [7]. Studies by Gomes et al. showed greater intraexaminer reliability for PPT examination of the masseter and temporalis muscles of control subjects, compared with TMD patients. Another study demonstrated that the repeatability of PPT is greater among asymptomatic patients than among those with painful conditions. In the same study, Gomes et al. suggested that algometry is likely to be useful in the identification of asymptomatic individuals, rather than for the evaluation of previously confirmed TMDs [4]. Among all masticatory muscles, Dos Santos Silva et al. found the lowest PPT value for the masseter and the highest for the posterior temporalis, consistent with other investigators' findings [8, 9]. Farella et al. found that, clinically, the pressure algometer may provide minimal aid in the examination of the most affected muscles; due to low positive predictive values, pressure algometry has limited use as a diagnostic tool [10]. Further, Haddad et al. investigated the correlation between thermography-assessed and clinical myofascial trigger points (MTPs) in masticatory muscles; they concluded that infrared imaging indicates differences between referred and local pain in MTPs. PPT values were higher for points of local pain than for points of heterotopic pain, which might be valuable in the identification of the pain source [9]. Studies comparing PPTs in healthy individuals and TMD patients showed significantly higher pain sensitivity and general hyperalgesia for TMD patients [10–18]. Gracely et al. found that individuals with chronic TMD were more pain-sensitive than patients with persistent and transient TMD [19].

Ayesh et al. found no sex-related differences [14, 20], while Oono et al. found significantly higher PPT values at baseline in men than in women [21]. Slade et al. revealed that PPT was connected with the course of painful TMD and remained lowered in persistent TMD, in comparison with transient TMD. They also found that PPT had no clinically useful prognostic value in predicting future development of TMD [19]. In a study of patients with anterior disc displacement without reduction, Craane et al. concluded that there was no influence on reduction of TMD symptoms between patients in an additional physical therapy program, in comparison with patients who received only information and instructions regarding TMD therapy [22]. Moreover, De Laat et al. reported that both counselling and physical therapy reduced myofascial pain within the masticatory system [23].

Farella et al. investigated masticatory muscle pain during, immediately after, and 1 day after sustained muscle contraction in a group of healthy participants. They found a reduced PPT of the jaw muscles only after long-lasting and low-level effort [24]. In contrast, Takeuchi et al. discovered that tooth clenching did not affect the masseter or temporalis muscle PPT in healthy individuals [25]. In turn, the research conducted by Cioffi et al. stated that occlusal interference in female volunteers with masticatory muscle pain did not affect the PPT of the masseter and temporalis muscle [16]. De Moraes et al. showed an increase in the masseter PPT after low-level laser therapy treatment (LLLT), which lasted 30 more days. The PPT elevation for the temporal muscle occurred only at the end of the treatment and was not sustained at 30 days thereafter. The placebo group did not show any change in PPT values throughout the study [12]. Öz et al. confirmed the previous conclusion that the PPT increases after LLLT [26]. Controversially, Magri et al. showed no difference after LLLT in the PPT values of the experimental groups [27].

In another study, Farella et al. examined changes in the PPT of the jaw muscles after orthognathic surgery for class III malocclusion. After surgery, PPTs of the masseter and temporalis muscles did not change significantly from baseline values, and there was no clear connection between surgery and the PPT [28]. Costa et al. showed that patients with chronic tension-type headaches had low PPT values at all examined points of the head, especially at the anterior temporalis muscle. Moreover, the presence of a headache did not influence the reduction of facial pain intensity, and there was no influence of a TMD-attributed headache on muscle pain [18]. The use of an occlusal appliance was investigated by Nilner et al. [29], who concluded that such usage increases PPT values for the right side of the masseter and both sides of the anterior temporalis, consistent with the findings of Öz et al. [26].

Vignolo et al. investigated the influence of menstrual cycle on the PPT of the masticatory muscle. They found that the menstrual phase did not influence PPT, but oral contraceptive use raised PPT values [15]. Conversely, Isselée et al. reported a statistically significant difference between menstrual cycle phases, regardless of oral contraceptive use. There were similar patterns of PPT values for the masseter, temporalis, and thumb muscles, with good long-term consistency in both males and females. However, PPTs of all muscles were significantly lower during perimenstrual phases in the female groups [30]. Dos Santos Silva et al. [8] found the highest sensitivity (77%) and probability ratio for TMD in the anterior masseter muscle. Von Piekartz et al. used an analogous study design and an algometer as a measuring tool. They assessed PPT as 0.35 kgf/cm^2^ (±0.47) for the right and 0.98 kgf/cm^2^ (±1.0) for the left masseter of an average TMD patient [31], significantly lower than in the study by Dos Santos Silva et al. [8]. Bernhardt et al. used a fingertip-shaped pressure algometer (PAP) and the Somedic algometer. Both showed high overall reliability and equally high capacity for differentiating TMD cases from controls. In that study, PAP yielded a significantly higher PPT than the Somedic algometer [32]. Silveira et al. found that elevated levels of muscle tenderness were associated with the severity of jaw and neck disabilities. Further, jaw dysfunction and neck disability were clearly correlated, such that fluctuations in jaw dysfunction may be explained by changes in neck disability and vice versa in TMD patients. That study emphasised the importance of assessing TMD at both the level of the jaw and the neck area. Pain and muscle sensitivity are a subset of TMD features. Notably, TMD provides a complex challenge that involves numerous factors, such as gender, stress exposure, and levels of anxiety [33]. Ferreira et al. investigated the influence of TENS (transcutaneous electrical nerve stimulation) on the PPT of masticatory muscles and found significantly higher PPT of the anterior temporalis, masseter, and sternocleidomastoid muscle in the active TENS group, compared with placebo. They concluded that TENS increased short-term PPT values [34]. In 2017, Costa et al. published additional research supporting their previous findings: tension-type headache patients have lower PPT values than nonheadache patients, particularly with respect to the anterior temporalis [35].

### 3.5. Risk of Bias across Studies

Generally, the risk of bias among individual studies was low to unclear. Since those biases were likely to lower the confidence in conclusions, we had to downgrade the levels of evidence by 2 points. Nevertheless, evidence from the clinical trials is still of high quality according to the GRADE approach.

## 4. Discussion

### 4.1. Summary of Evidence

Among the studies covered by 32 full-text articles, 11 used the Somedic algometer, four used the Wagner algometer, six used the Kratos digital dynamometer, and seven used a variety of other models. Four studies did not describe the exact model of the device used. We also found discrepancies regarding the pressure increase rate. In nine studies, the pressure increased at a rate of 30 kPa/s, in three at a rate of 40 kPa/s, in three at a rate 20 kPa/s, and twelve used 50 kPa/s. Pressure increase values grow from 20 kPa/s to 50 kPa/s and from 0.5 kg/cm^2^/s to 2 kg/cm^2^/s. In five articles, the pressure increase rate was not mentioned.

Descriptions of the PPT examination points also varied, from very precise and repeatable to a simple listing of anatomical points (e.g., the whole muscle without indicating the exact location). The number of measurements, intervals, and relevance of each examination also differed among the respective studies. The number of measurements varied from 1 to 5 at each visit. The intervals ranged from 5 seconds to 15 minutes. Some studies argued that the first measurement was not valid because of its higher numeric value and did not use it for further analysis, while others used all results to calculate average values. In terms of the presence of the control point for the PPT measurement outside the area under examination, the authors found that the majority of the studies (13 articles) did not meet this condition.

Most articles had similar exclusion criteria which were as follows:

- Orofacial trauma
- Systemic disorders
- Cervical disorders (pain, pain upon movement within range of cervical spine)
- Neurologic disorders
- Drug or alcohol abuse
- Use of antidepressants, hormonal medications, muscle relaxants, and painkillers
- Wearing orthodontic braces
- More than five headaches per month in the 3 months before enrolment
- Evoked pain in more than three muscle locations (myalgia) or in more than one tmj (arthralgia)
- Complaints suggesting episodic neuropathic pain
- Pregnancy
- Missing more than two posterior teeth
- Presence of full or removable partial denture
- Severe malocclusion (overbite and overjet > 6 mm)
- Unilateral or anterior crossbite
- Discrepancy of centric relation to maximum intercuspation > 5 mm
- Rheumatoid diseases
- TMD treatment performed in the last three months

There were exceptions according to the exclusion criteria if a study investigated patients with respect to the connection between headache and TMD or the long-term effects of orthognathic surgery.

### 4.2. Limitations

This systematic review has some underlying limitations. Most importantly, the articles lack in methodological homogeneity regarding pressure increase rate, the number of measurements, and measurement intervals. Additionally, different algometers were used in the respective studies.

### 4.3. Conclusions

Overall, based on the analyzed articles, the authors state that the PPT is not an efficient approach for screening and evaluating orofacial pain. What is more, it should not be used as the only diagnosis for patients with orofacial pain. The papers identified for this review lack consistency in terms of study design and patient management. Many additional factors should be considered in the future prior to evaluation of PPTs (e.g., body symmetry and posture, hormone levels and menstrual phases in women). Notably, medication use is a growing factor in the ageing society, such that its influence on the PPT should also be thoroughly scrutinised. Further research is required regarding other treatment interventions, such as splint therapy, or combinations of specialised physical or pharmacotherapy. As some studies have confirmed, the pressure algometer is an efficient and effective tool in screening and evaluating orofacial pain patients [5, 14, 15, 21, 32]. Importantly, however, additional well-designed clinical trials of the PPT are needed, involving larger groups of orofacial pain patients, as they should greatly benefit from the unification of procedures, examination points, and devices used, combined with digitalization of the entire process. This might lead to the development of new PPT examination standards for pain practice.

## Figures and Tables

**Table 1:** Assessment of risk of bias.

	RSG^1^	AC^2^	BoPaP^3^	BoOA^4^	IOD^5^	SR^6^	OB^7^
Chaves 2010	+	−	−	−	+	+	+
Chaves 2013	+	+	+	+	+	+	+
Visscher 2004	+	−	+	+	?	?	+
Gomes 2014	+	−	+	−	+	+	−
Silva 2005	−	−	+	−	?	?	−
Haddad 2012	−	−	−	−	?	?	+
Farella 2000	−	−	+	+	?	?	−
Maia 2012	+	+	+	−	−	−	+
Ayesh 2007	−	−	−	−	+	+	−
Vignolo 2008	−	−	−	−	?	?	−
Cioffi 2015	+	+	+	+	−	−	+
Silveira 2014	−	−	+	+	?	?	?
Costa 2015	−	−	+	+	−	−	−
Ayesh 2006	−	−	−	+	?	?	+
Oono 2012	−	−	−	+	+	+	+
Craane 2012	+	+	+	+	−	−	+
Laat 2003	+	+	+	+	−	−	+
Öz 2010	+	+	+	+	−	−	+
Magri 2017	+	+	+	+	+	+	+
Nilner 2008	+	+	+	+	−	−	−
Isleé 2001	−	−	−	−	?	?	?
Piekartz 2014	+	+	+	+	−	−	−
Bernhardt 2007	−	−	−	−	?	?	+
Silveira 2014	−	−	+	−	?	?	+
Ferreira 2017	+	+	+	−	+	+	+

^1^Random sequence generation, ^2^allocation concealment, ^3^blinding of participants and personnel, ^4^blinding of outcome assessment, ^5^incomplete outcome data, ^6^selective reporting, and ^7^other bias. Risk of bias high (−), risk of bias low (+), and risk of bias unknown (?).
